# Rapid onset vasodilation with single muscle contractions in the leg: influence of age

**DOI:** 10.14814/phy2.12516

**Published:** 2015-08-28

**Authors:** William E Hughes, Kenichi Ueda, David P Treichler, Darren P Casey

**Affiliations:** 1 Department of Physical Therapy and Rehabilitation Science, Carver College of Medicine, University of IowaIowa City, Iowa; 2 Department of Anesthesia, Carver College of Medicine, University of IowaIowa City, Iowa

**Keywords:** Aging, exercise, hyperemia, limb, vasodilation

## Abstract

The influence of aging on contraction-induced rapid vasodilation has been well characterized in the forearm. We sought to examine the impact of aging on contraction-induced rapid vasodilation in the leg following single muscle contractions and determine whether potential age-related impairments were similar between limbs (leg vs. arm). Fourteen young (23 ± 1 years) and 16 older (66 ± 1 years) adults performed single leg knee extensions at 20%, 40%, and 60% of work rate maximum. Femoral artery diameter and blood velocity were measured using Doppler ultrasound. Limb vascular conductance (VC) was calculated using blood flow (mL·min^−1^) and mean arterial pressure (mmHg). Peak and total vasodilator responses in the leg (change [Δ] in VC from baseline) were blunted in older adults by 44–50% across exercise intensities (*P *<* *0.05 for all). When normalized for muscle mass, age-related differences were still evident (*P *<* *0.05). Comparing the rapid vasodilator responses between the arm and the leg of the same individuals at similar relative intensities (20% and 40%) reveals that aging influences peak and total vasodilation equally between the limbs (no significant age × limb interaction at either intensity, *P *=* *0.28–0.80). Our data demonstrate that (1) older adults exhibit an attenuated rapid hyperemic and vasodilator response in the leg; and (2) the age-related reductions in rapid vasodilation are similar between the arm and the leg. The mechanisms contributing to the age-related differences in contraction-induced rapid vasodilation are perhaps similar to those seen with the forearm model, but have not been confirmed.

## Introduction

At the onset of exercise there is an obligate, rapid rise in blood flow and vasodilation, ultimately contributing to the fine-tuning of exercise hyperemia and meeting the metabolic demands of contracting tissue during steady-state exercise. The immediate exercise hyperemia and vasodilation following a single muscle contraction tends to peak within approximately five cardiac cycles postcontraction, is graded with exercise intensity, and has been termed “rapid onset vasodilation” (Tschakovsky et al. 1996, 2004; Naik et al. 1999; Clifford 2007). The rapid vasodilation following a contraction is inherently linked to steady-state blood flow, acting as a feedforward mechanism, contributing to the rapid transition between exercise workloads (Shoemaker and Hughson 1999; Saunders and Tschakovsky 2004). The local regulation of contraction-induced rapid vasodilation has been attributed to a complex interplay between endothelial (Armstrong et al. 2007; Casey et al. 2013; Crecelius et al. 2013), mechanical (Tschakovsky et al. 1996; Kirby et al. 2007), and adrenergic factors (Jackson et al. 2010; Casey and Joyner 2012), with no one factor having an obligatory role in this immediate hyperemic and vasodilator response.

In humans the majority of the evidence for contraction-induced rapid vasodilation has been characterized in the forearm. Collectively, this evidence has shown that older adults exhibit an attenuated rapid vasodilator response compared to young adults, which may be due to a diminished role of nitric oxide (NO) signaling, enhanced *α*-adrenergic vasoconstrictor tone and/or a reduced ability to blunt sympathetic vasoconstriction (Casey and Joyner 2012; Casey et al. 2013). While these studies contribute to the knowledge of local regulation of blood flow and vasodilation, it is not known if age-related impairments in contraction-induced rapid vasodilation can be generalized to the lower limbs. Recent evidence suggests that rapid vasodilation following a single isometric contraction is observed in the leg of young men, and the magnitude of this response is similar between the arm and the leg (Credeur et al. 2015). However, it is unclear how aging influences contraction-induced rapid vasodilation in the leg. In this context, the lower limbs are subject to greater hydrostatic forces (i.e., gravity), and locomotion presents a constant hemodynamic challenge to the leg vasculature (Rowell 1993). Moreover, evidence suggests that limb-specific differences in vasodilator responsiveness exist with aging. That is, the blood flow and vasodilator responses to exogenous vasodilators, limb ischemia, and steady-state exercise in the leg of older individuals are attenuated to a greater extent relative to the forearm (Newcomer et al. 2004; Proctor et al. 2005; Ridout et al. 2005; Donato et al. 2006; Proctor and Newcomer 2006; Nishiyama et al. 2008). Furthermore, the lower limbs have a greater propensity to develop atherosclerotic lesions, and have a higher prevalence of peripheral arterial disease (Walden et al. 1985). Collectively, these findings suggest that older adults would exhibit an impairment of contraction-induced rapid vasodilation in the leg, and this impairment is potentially greater than what has been demonstrated in the forearm (Carlson et al. 2008; Kirby et al. 2009; Casey and Joyner 2012; Casey et al. 2013). Therefore, the primary purpose of this study was to characterize the effects of aging on contraction-induced rapid vasodilation in the leg. Additionally, we aimed to examine whether potential age-related impairments in rapid vasodilation following a single muscle contraction were greater in the leg compared to the forearm.

## Methods

### Subjects

A total of 14 young (9 men and 5 women, age: 20–26 years) and 16 older (10 men and 6 women, age: 60–72 years) subjects volunteered to participate in this study. Subjects completed a general health history screening and written informed consent. Subjects were generally healthy, nonobese (body mass index ≤30 kg/m^2^), nonsmokers, not taking any vasoactive medications, and were self-reported as sedentary to recreationally active, with no regular physical training. Studies were performed after an overnight fast, and subjects refrained from exercise, alcohol, and caffeine for 24 h before reporting to the laboratory. Young female subjects were studied during the early follicular phase of their menstrual cycle or the placebo phase of oral contraceptives to control for the potential influence of sex hormones on primary outcome variables (Minson et al. 2000; Chan et al. 2001). All older female subjects were postmenopausal and were not taking any form of hormone replacement therapy. All study protocols were approved by the Institutional Review Board at the University of Iowa.

### Body composition, forearm, and leg tissue mass

Body composition was determined by dual energy X-ray absorptiometry (DEXA; Hologic software version APEX 4.0). Total mass and fat-free mass of the left forearm and right leg were determined from regional analysis from the whole body DEXA scan using bony landmarks for normalization of blood flow and vascular conductance responses for between-group and between-limb comparisons. Body mass index was calculated as body weight (kg) divided by height (meters) squared.

### Heart rate and systemic blood pressure

Heart rate (HR) was recorded via continuous three-lead electrocardiogram, and systemic blood pressure was assessed (beat-to-beat) via finger plethysmography (Nexfin; Edwards Lifesciences, Irvine, CA) on the nonexercising hand. Brachial artery pressure was measured in duplicate using an automated cuff (Cardiocap/5, Datex-Ohmeda, Louisville, CO) prior to beginning exercise trials while the subjects were in a supine position following 15 min of rest.

### Prestudy day measurements: determination of work rate maximum

Work rate maximum (WR_max_) was determined from a single leg knee extensor incremental maximal exercise test completed during a familiarization session prior to the study day. Subjects were seated in a semirecumbent position on a modified adjustable bucket seat that accommodated variable body and leg lengths allowing each subject’s lower leg to move through a 90–180 degree range of motion during the knee extension exercise. Both knees were flexed at 90 degrees with a form fitting orthopedic boot attached to the right ankle. The boot was attached to a leg shaft located behind the knee that had a one-way clutch bearing that allowed for no resistance as the leg returns to 90 degrees flexion (eccentric). Resistance was developed by contracting (concentric) against the leg shaft with the device electronically developing the torque. WR_max_ testing consisted of an initial workload of 5 W that incrementally increased every minute by 3 W and 5 W in female and male subjects, respectively. Subjects kicked dynamically through a full range of motion at a cadence of 40 kicks per minute. The single leg knee extensor incremental maximal test continued until the subject could not maintain a full range of motion or 40 contractions per minute. The final workload completed was recorded as maximal kicking load from which relative workloads were calculated (Limberg et al. 2010).

### Study day: single muscle contractions

Subjects performed single knee extension contractions on the custom-made, computer-controlled leg ergometer (described above) at 20%, 40%, and 60% WR_max_ with the order of exercise intensities randomized across subjects. Subjects were instructed to contract and relax on a verbal command from laboratory personnel. To compare rapid hyperemic and dilator responses between limbs (leg vs. arm), single forearm contractions were performed with a handgrip device at 10%, 20%, and 40% of the subject’s maximal voluntary contraction (MVC), determined (using a handgrip dynamometer) as the average of three maximal squeezes performed on the prestudy measurement day. While supine, the weight for each respective exercise intensity was lifted 4–5 cm over a pulley for a single, 1-sec muscle contraction. WR_max_ and MVC intensities were randomized prior to the experimental protocol and each contraction intensity was performed in duplicate to calculate the average response for each subject for a given condition. Each contraction (leg and arm) was visually observed by the laboratory personnel to ensure proper timing of contraction and 2 min of relaxation were given between each contraction to allow continuous measures of limb hemodynamics postcontraction. All single muscle contractions (knee extensions and forearm contractions) were performed on the same day.

### Measurement of blood flow

Common femoral (∼2–3 cm proximal to bifurcation) and brachial artery diameter and blood velocity were determined with a 12-MHz linear-array Doppler probe (model M12L; Vivid 7, General Electric, Milwaukee, WI). Blood velocity was measured with a probe insonation angle previously calibrated to 60 degrees. Measured velocity waveforms were synchronized to a data acquisition system (WinDaq; DATAQ Instruments, Akron, OH) via a Doppler audio transformer (Herr et al. 2010). Artery diameter measurements were obtained at end diastole at rest (before contraction) and 1 min postcontraction. Limb blood flow (BF) was calculated as the product of mean blood velocity (cm/sec) and artery cross-sectional area (cm^2^) and expressed as milliliters per minute (mL·min^−1^).

### Data analysis and statistics

Data were collected at 250 Hz and analyzed offline with signal processing software (WinDaq; DATAQ Instruments). Mean arterial pressure (MAP) was derived from the Nexfin pressure waveform and HR was determined from the electrocardiogram. Baseline BF and MAP represent an average of the last 30 sec of the resting time period before each muscle contraction and were used to quantify the hyperemic response. Vascular conductance (VC) was calculated as BF/MAP × 100 (and expressed as mL·min^−1^·100 mmHg^−1^). Rapid hyperemic and vasodilator responses are expressed as the change in (Δ) BF and VC, respectively. Of particular interest are the immediate (i.e., first cardiac cycle postcontraction), peak, and total dilator responses postcontraction. Total BF (mL) and VC (mL·100 mmHg^−1^) were defined as the area under the curve after respective baseline values were subtracted for a given flow or conductance curve. To account for the possible influence of muscle mass, BF and VC were normalized to muscle mass (kg) (Donato et al. 2006; Parker et al. 2007).

All values are expressed as mean ± SE. Analysis of variance (ANOVA) was used to analyze demographic variables between age groups. To address the primary question of whether contraction-induced rapid vasodilation is impaired in the leg with aging, a two-way repeated measures ANOVA was used to evaluate leg blood flow and vasodilator differences between age groups (young vs. older) across exercise intensities (20%, 40%, and 60%). To compare responses between limbs, BF and VC at 20% and 40% MVC and WR_max_ were analyzed via independent two-way ANOVA (20% and 40%). When significance was detected, Tukey’s post hoc analysis was used to identify differences between groups. All statistical analyses were completed using SigmaStat software version 12.0 (Systat Software Inc., San Jose, CA). Statistical difference was set a priori at *P *<* *0.05.

## Results

Subject characteristics are shown in Table1. Young and older subjects were of similar height, weight, and body mass (*P *>* *0.05), but older adults had a higher percent body fat (*P *<* *0.05). Additionally, young and older subjects exhibited similar brachial blood pressures, forearm and leg muscle masses, and forearm MVC (*P *>* *0.05), but WR_max_ in the leg was lower in the older adults (*P *<* *0.05).

**Table 1:** Subject characteristics

Variable	Young adults (*n* = 14)	Older adults (*n* = 16)
Age (years)	23 ± 1	66 ± 1
Men/Women	9/5	10/6
Height (cm)	174 ± 2	172 ± 2
Weight (kg)	75 ± 3	78 ± 3
Body mass index (kg·m^2^)	24.7 ± 0.6	26.5 ± 0.7
% Body fat	27.1 ± 1.9	31.9 ± 1.7*
Forearm muscle mass (kg)	0.94 ± 0.08	0.90 ± 0.06
Thigh muscle mass (kg)	7.5 ± 0.5	6.9 ± 0.3
MVC (kg)	41 ± 3	41 ± 3
WR_max_ (W)	41 ± 4	29 ± 2*
Systolic BP (mmHg)	117 ± 2	123 ± 3
Diastolic BP (mmHg)	72 ± 2	75 ± 2
MAP (mmHg)	87 ± 2	91 ± 2

Values are mean ± SE.

MVC, maximal voluntary contraction; WR_max_, work rate maximum.

* *P* < 0.05 vs. young adults.

### Characterizing ROV in the leg with aging

**Figure 1:**
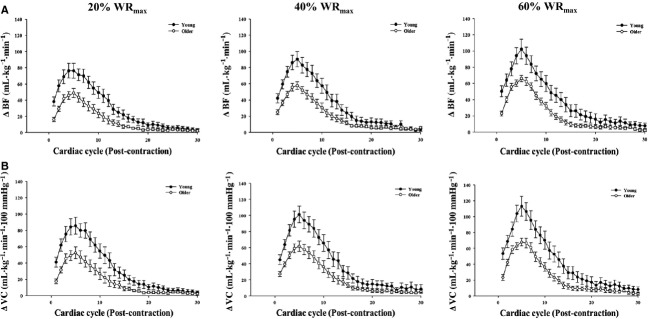
(A) Hyperemic (change [Δ] in blood flow [BF]) and (B) vasodilator (Δ vascular conductance [VC]) responses over 30 cardiac cycles following single leg extension contractions at 20%, 40%, and 60% WR_max_.

**Figure 2:**
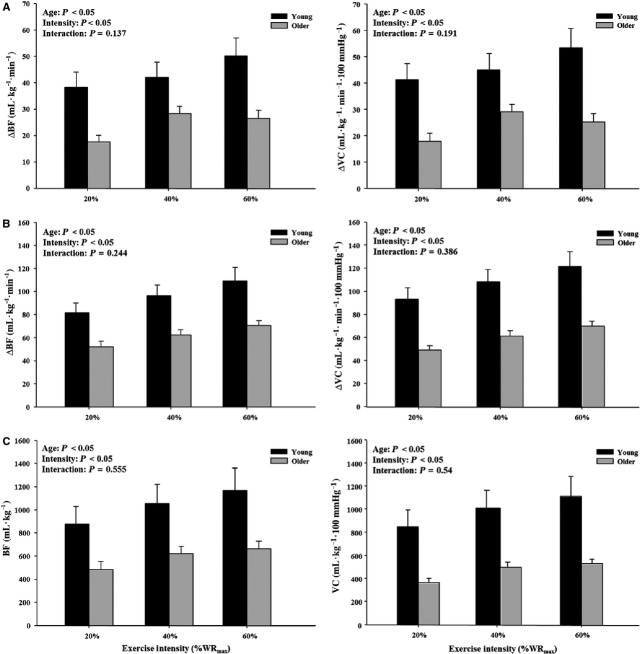
(A) Immediate (first cardiac cycle postcontraction), (B) peak, and (C) total hyperemic (ΔBF) and vasodilator (ΔVC) responses normalized for muscle mass to single leg extension contractions between young and older adults at 20%, 40%, and 60% WR_max_. All parameters of the ROV response (immediate, peak, and total) were blunted in older adults across each workload.

Baseline (i.e., resting) BF, MAP, and VC did not differ with age across each trial (Table2). When expressed as the absolute change from baseline, the immediate, peak, and total hyperemic and vasodilator responses following single knee extension contractions were attenuated in older adults across exercise intensities (Table3; *P *<* *0.05). Similarly, the relative change from baseline in the immediate and peak hyperemic and vasodilator responses were also blunted in the older compared to young adults at each workload (Table3; *P *<* *0.05). Figure1 illustrates the time course for the absolute rapid hyperemic and vasodilator responses normalized for muscle mass following single knee extension contractions at 20%, 40%, and 60% WR_max_ in young and older adults. The immediate, peak, and total hyperemic and vasodilator responses (normalized for muscle mass) were substantially attenuated in the older adults across exercise intensities (*P *<* *0.05; Fig.2A–C). Moreover, when the relative change in immediate and peak hyperemia and vasodilation normalized for muscle mass was examined, older adults still exhibited lower responses (data not shown).

**Table 2:** Baseline hemodynamics under each condition

Leg									
	Young			Older			Factor *P*-values		
	20% WR_max_	40% WR_max_	60% WR_max_	20% WR_max_	40% WR_max_	60% WR_max_	Age	Intensity	Interaction
Diameter (cm)	0.89 ± 0.03	0.89 ± 0.03	0.89 ± 0.03	0.95 ± 0.03	0.97 ± 0.03	0.96 ± 0.03	<0.05	0.97	0.98
MAP (mmHg)	94 ± 3	95 ± 3	95 ± 3	98 ± 3	98 ± 2	99 ± 2	0.06	0.92	0.99
BF (mL·min^−1^)	172 ± 13	173 ± 14	167 ± 13	175 ± 20	176 ± 16	180 ± 18	0.64	0.99	0.94
VC (mL·min^−1^·100 mmHg^−1^)	184 ± 14	171 ± 15	177 ± 14	179 ± 19	180 ± 17	182 ± 18	0.92	0.99	0.94
HR (bpm)	65 ± 3	65 ± 3	64 ± 3	61 ± 1	61 ± 1	62 ± 1	0.06	0.99	0.89

Arm									
	Young			Older					
	10% MVC	20% MVC	40% MVC	10% MVC	20% MVC	40% MVC			
Diameter (cm)	0.37 ± 0.02	0.37 ± 0.02	0.37 ± 0.02	0.41 ± 0.02	0.41 ± 0.02	0.41 ± 0.02	<0.05	0.99	0.99
MAP (mmHg)	89 ± 3	89 ± 3	89 ± 3	95 ± 2	95 ± 2	95 ± 2	<0.05	0.98	0.99
BF (mL·min^−1^)	38 ± 6	39 ± 6	41 ± 6	47 ± 3	50 ± 4	52 ± 4	<0.05	0.66	0.98
VC (mL·min^−1^·100 mmHg^−1^)	45 ± 8	46 ± 8	48 ± 8	50 ± 3	53 ± 4	55 ± 4	0.17	0.78	0.98
HR (bpm)	64 ± 3	63 ± 2	63 ± 3	64 ± 2	64 ± 2	65 ± 2	0.68	0.98	0.95

Values are mean ± SE.

MAP, mean arterial pressure; BF, blood flow; VC, vascular conductance; HR, heart rate; WR_max_, work rate maximum; MVC, maximal voluntary contraction.

**Table 3:** Comparison of absolute and relative blood flow and vascular conductance not normalized for muscle mass

	Young adults			Older adults			Factor *P*-values		
	20% WR_max_	40% WR_max_	60% WR_max_	20% WR_max_	40% WR_max_	60% WR_max_	Age	Intensity	Interaction
**Absolute responses**									
Δ Blood flow (mL·min^−1^)									
Immediate	285 ± 39	320 ± 47	373 ± 48	118 ± 18	195 ± 21	181 ± 22	<0.05	<0.05	0.18
Peak	598 ± 57	724 ± 68	802 ± 83	354 ± 27	431 ± 34	485 ± 29	<0.05	<0.05	0.14
Total	6249 ± 1007	7737 ± 1013	8371 ± 1224	3219 ± 395	4273 ± 456	4557 ± 417	<0.05	<0.05	0.47
Δ Vascular conductance (mL·min^−1^·100 mmHg^−1^)									
Immediate	308 ± 43	343 ± 52	400 ± 57	121 ± 16	201 ± 19	186 ± 23	<0.05	<0.05	0.24
Peak	690 ± 65	818 ± 79	900 ± 99	373 ± 34	456 ± 41	504 ± 33	<0.05	<0.05	0.26
Total	7108 ± 1151	8715 ± 1148	9439 ± 1329	3501 ± 474	4612 ± 534	4825 ± 483	<0.05	<0.05	0.46
**Relative responses**									
% Δ Blood flow (mL·min^−1^)									
Immediate	170 ± 21	189 ± 26	223 ± 20	75 ± 14	119 ± 12	118 ± 19	<0.05	<0.05	0.28
Peak	356 ± 28	422 ± 30	488 ± 34	221 ± 24	272 ± 29	308 ± 30	<0.05	<0.05	0.19
% Δ Vascular conductance (mL·min^−1^·100 mmHg^−1^)									
Immediate	172 ± 22	179 ± 30	222 ± 21	75 ± 13	120 ± 11	120 ± 19	<0.05	<0.05	0.17
Peak	384 ± 30	449 ± 34	512 ± 36	223 ± 24	277 ± 29	323 ± 29	<0.05	<0.05	0.41

Values are mean ± SE.

WR_max_, work rate maximum.

### Examination of potential limb differences with aging

**Figure 3:**
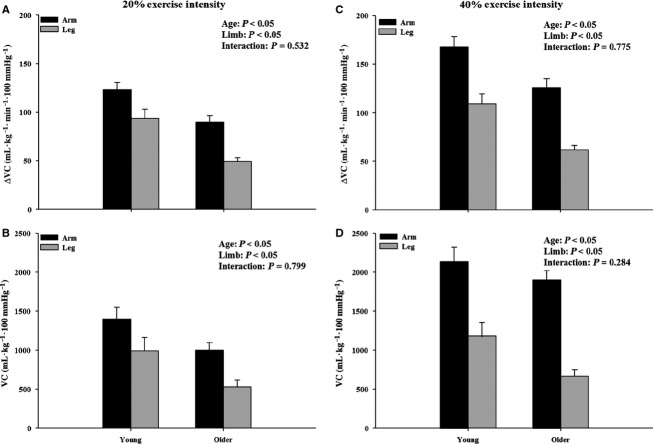
Peak and total ΔVC normalized for muscle mass at (A, B) 20% and (C, D) 40% exercise intensity in the arm and the leg. Peak and total ΔVC were blunted in the arm and the leg of older adults at both exercise intensities.

Table4 shows all parameters of absolute rapid vasodilation (immediate, peak, and total) at both 20% and 40% exercise intensity. At the 20% exercise intensity all parameters of absolute rapid vasodilation (immediate, peak and total) were substantially greater in the leg compared to the forearm in both young and older adults. To account for the inherent volume and muscle mass differences between the thigh and forearm, we also compared the vasodilator responses normalized for muscle mass in each respective limb. Utilizing this approach revealed a significant main effect of limb on the peak and total vasodilator response at 20% and 40% exercise intensity (*P *<* *0.05). However, when normalized for muscle mass, greater vasodilator responses were observed in the forearm (Fig.3). Of particular interest to the current study, there was also a main effect of age but not a significant age × limb interaction. That is, despite older adults having lower vasodilator responses compared to their young counterparts, the degree of attenuation was similar between the arm and the leg (Fig.3).

**Table 4:** Comparison of absolute change in rapid vasodilation in the arm and leg of young and older adults at similar intensities

	Young		Older		Factor *P*-values		
	Δ Leg VC (mL·min^−1^·100 mmHg^−1^)	Δ Arm VC (mL·min^−1^·100 mmHg^−1^)	Δ Leg VC (mL·min^−1^·100 mmHg^−1^)	Δ Arm VC (mL·min^−1^·100 mmHg^−1^)	Age	Limb	Interaction
20% Workload							
Immediate	308 ± 43*†	44 ± 6	121 ± 18*	35 ± 3	<0.05	<0.05	<0.05
Peak	690 ± 65*†	117 ± 12	373 ± 34*	83 ± 6	<0.05	<0.05	<0.05
Total	7108 ± 1151*†	1289 ± 140	3501 ± 474*	870 ± 73	<0.05	<0.05	<0.05
40% Workload							
Immediate	343 ± 52*†	69 ± 6	201 ± 19*	52 ± 5	<0.05	<0.05	<0.05
Peak	818 ± 79*†	159 ± 17	456 ± 41*	112 ± 7	<0.05	<0.05	<0.05
Total	8715 ± 1148*†	2013 ± 235	4612 ± 534*	1675 ± 124	<0.05	<0.05	<0.05

Values are mean ± SE.

VC, vascular conductance; 20% workload, WR_max_ (leg), MVC (arm); 40% workload, WR_max_ (leg), MVC (arm).

* *P *<* *0.05 vs. arm.

† *P *<* *0.05 vs. older adults.

## Discussion

Previous studies by our group, as well as others, have demonstrated that contraction-induced rapid vasodilation is blunted in the forearm of older adults (Carlson et al. 2008; Kirby et al. 2009; Casey and Joyner 2012; Casey et al. 2013). In the present study, we examined whether age-related impairments in the hyperemic and vasodilator response to a single muscle contraction are also present in the leg. Furthermore, we aimed to examine whether the potential age-related reduction in leg vasodilation following a single muscle contraction is greater than that seen in the forearm. Our primary novel findings are 1) older adults exhibit a substantially attenuated rapid hyperemic and vasodilator response in the leg and 2) the age-related reductions in rapid vasodilation appear to be similar between the arm and the leg.

To the best of our knowledge, only one other study has examined rapid vasodilation after a single skeletal muscle contraction in the leg. Credeur and colleagues (2015) demonstrated that in young men, a single isometric contraction in the leg produced an intensity-dependent increase in blood flow and vasodilation, similar to that seen in the forearm. They also found that the vasodilator response to single isometric contractions was influenced by body position (greater when leg was positioned below heart level), and largely independent of mechanical factors. The results in young adults from our current study are in agreement with Credeur et al. (2015), in that a single dynamic muscle contraction in the leg (knee extension) produced an intensity-dependent increase in blood flow and vascular conductance. The novelty of our current findings is that the rapid hyperemic and vasodilator response following a dynamic muscle contraction in the leg is substantially attenuated in older adults across a range of intensities. Additionally, the blunting of rapid vasodilation in the leg with aging is evident regardless of expressing the response in absolute or relative terms (Table3) and is independent of muscle mass (Fig.2).

Similar to previous reports (Donato et al. 2006; Wray et al. 2009), the older adults in the present study had a lower WR_max_ compared to their young counterparts (Table1) and consequently performed single contractions at a lower absolute workload in each trial. Therefore, it could be argued that the blunted rapid vasodilation in the older adults is simply due to a lower absolute workload performed at each relative intensity. However, close examination of our data suggest this is not likely the case. When we compare the hyperemic and vasodilator responses in the older adults at 60% WR_max_ (∼17 W) to that of the young adults at 40% WR_max_ (∼16 W), there is still a significant age-related difference in the immediate, peak, and total BF and VC (expressed for both absolute and normalized for muscle mass). Additionally, if we were to match a subset of the subjects (*n *=* *9 for each age group) for identical workloads within each relative exercise intensity and effectively eliminate any difference in WR_max_ between age groups, the attenuated hyperemic and vasodilator responses in older adults still persist. This is true for the immediate, peak, and total BF and VC and is independent on whether the response is quantified in absolute terms or normalized for muscle mass.

There is strong evidence that aging is associated with a wide number of modifications to arterial structure and function (Lakatta and Levy 2003; Najjar et al. 2005). Moreover, the control of blood flow to dynamically contracting skeletal muscle is altered with aging during submaximal exercise (Proctor et al. 1998, 2003; Poole et al. 2003; Donato et al. 2006; Schrage et al. 2007; Kirby et al. 2009). Studies in both animals and humans suggest that the age-related impairments in the control of muscle blood flow are apparent at the very onset of exercise (i.e., as early as the first contraction) (Carlson et al. 2008; Kirby et al. 2009; Jackson et al. 2010; Casey and Joyner 2012; Casey et al. 2013). To date, all evidence for age-related differences in rapid onset vasodilation in humans has been restricted to the forearm as a model (Carlson et al. 2008; Kirby et al. 2009; Casey and Joyner 2012; Casey et al. 2013). Nonetheless, findings from these studies clearly demonstrate that the peak blood flow and vasodilator response to a single muscle contraction is significantly blunted in older compared to young adults.

Recent evidence from our laboratory has pointed to a role for reduced NO bioavailability and/or signaling as well as enhanced *α*-adrenergic vasoconstriction as possible mechanisms for the blunting of rapid vasodilation in the forearm with aging (Casey and Joyner 2012; Casey et al. 2013). Therefore, it is conceivable that the attenuated rapid vasodilation in the leg of older adults in the present study might also be due to alterations in each of or a combination of the aforementioned mechanisms. In support of this notion, endothelium-dependent vasodilation (as assessed by flow-mediated dilation) is reduced in the leg of older adults (Parker et al. 2006; Nishiyama et al. 2008), with some of the evidence suggesting that the age-related impairments are greater in the leg compared to the arm (Nishiyama et al. 2008). Moreover, the leg blood flow response to passive leg movement is severely blunted with aging and the contribution of NO to this response in older adults is significantly reduced (Trinity et al. 2015). Additionally, intra-arterial infusions of an *α*-adrenergic receptor antagonist (phentolamine) effectively removes sympathetic restraint in the forearm and abolishes the age-related differences in hyperemic and vasodilator responses to single muscle contractions (Casey and Joyner 2012). Although it is unclear whether sympathetic restraint of the rapid vasodilator response in the lower limbs exists, some studies suggest the legs demonstrate an enhanced vasoconstrictor tone and augmented adrenergic responsiveness compared to the forearm in young adults (Dinenno et al. 2001; Pawelczyk and Levine 2002). With respect to aging, older adults have a greater tonic vasoconstriction in the leg when compared to their young counterparts (Smith et al. 2007), however, the *α*-adrenergic vasoconstrictor responsiveness to agonist-mediated stimulation is reduced (Smith et al. 2007; Wray et al. 2009). Furthermore, during dynamic exercise, sympathetic vasoconstrictor responsiveness appears to be augmented in older men (Koch et al. 2003). Taken together with our current results, these previous findings suggest a possible role for NO-mediated mechanisms as well as enhanced *α*-adrenergic vasoconstriction in the leg of older adults as a possible explanation for the age-related differences in contraction-induced rapid vasodilation.

In the present study, we also examined whether the age-related blunting of rapid vasodilation in response to a muscle contraction is more pronounced in the leg compared to the forearm. In both young and older adults, the absolute change in all parameters of contraction-induced vasodilation (immediate, peak, and total) was greater in the leg as compared to the forearm at similar relative exercise intensities (20% and 40% MVC WR_max_ and MVC, respectively). The greater rapid vasodilation observed in the leg as compared to the forearm is in agreement with previous studies in young males (Credeur et al. 2015). However, these limb differences observed in both age groups of the current study may simply be a product of the leg having a greater volume and muscle mass than the forearm. To try to circumvent these inherent differences between limbs we normalized the vasodilator responses for the muscle mass of either the forearm or the thigh. This approach revealed a greater rapid vasodilator response in the forearm in both young and older adults (Fig.3). However, it should be noted that the muscle mass values derived via DEXA used to normalize the responses in the leg included the entire thigh. This in turn likely led to an overestimation of the muscle mass involved in the contraction. Therefore, the significantly lower rapid vasodilator responses observed in the leg after normalizing for muscle mass might be explained by the large denominator used in the calculation. Along these lines, when estimates of quadriceps muscle mass (via anthropometric methods) are used to normalize blood flow and vasodilator responses during steady-state exercise at similar relative intensities, the response is substantially greater in the leg compared to the forearm (Donato et al. 2006). Nonetheless, our results indicate that, regardless of how vasodilator responses are expressed (absolute or normalized for muscle mass), the age-related attenuation of contraction-induced rapid vasodilation appears to be similar between limbs.

### Experimental considerations

There are a few experimental considerations for the current study that warrant discussion. First, the type of muscle contraction performed in the forearm and leg differed slightly. That is, the single leg knee extensor model employed in the present study consisted of a passive relaxation (e.g., no resistance during the eccentric portion), whereas the weight was constant during the contraction and relaxation phases of the forearm contractions (no passive relaxation). Therefore, it is conceivable that the differences in the blood flow and vasodilator responses between limbs are confounded to some degree by the type of muscle contraction performed. Second, our present findings suggest that the age-related attenuation of contraction-induced rapid vasodilation appears to be similar between limbs. However, it should be noted that the older adults demonstrated a similar handgrip strength (MVC) as their young counterparts, but had a significantly (∼30%) lower exercise capacity (WR_max_) in the leg. Since relative workloads were used to examine the rapid vasodilator response of the arm and the leg, the discrepancy in strength/exercise capacity between limbs with aging may have masked a greater age-related deficit in rapid onset vasodilation in one limb versus the other.

Finally, in the current study we did not observe age-related differences in leg blood flow at rest (Table2). This is in contrast to several other studies that have demonstrated that older adults have lower resting leg blood flow (Dinenno et al. 1999; Moreau et al. 2003; Smith et al. 2007). However, it should be noted that these previous studies reflect supine blood flow, whereas the resting blood flow measurements in the present study were performed with the subjects in a semirecumbent position. Indeed, Donato et al. (Donato et al. 2006) also reported that resting blood flow is similar in upright seated young and older adults. They speculated that the equal resting leg blood flow and vascular conductance between age groups while seated was potentially due to a pronounced increase in leg vascular tone in the young adults, while older individuals have a diminished response while in an upright position.

## Conclusion

To the best of our knowledge, this is the first study to demonstrate that aging blunts contraction-induced rapid vasodilation in the leg across exercise intensities and is largely independent of muscle mass. These findings are in agreement with previous studies demonstrating a reduced rapid vasodilator response in the forearm of older adults (Carlson et al. 2008; Kirby et al. 2009; Casey and Joyner 2012; Casey et al. 2013). Our current data also suggest that age-related impairments in contraction-induced vasodilation do not appear to be different between limbs (arm vs. leg). The primary mechanism(s) and their interactions which contribute to the substantially attenuated rapid hyperemic and vasodilator response in the leg of older adults remains unknown, but due to similar age-related reductions in contraction-induced rapid vasodilation between the leg and arm, it can be hypothesized that the mechanisms might be similar between limbs.
